# Seroprevalence, correlates and kinetics of SARS-CoV-2 nucleocapsid IgG antibody in healthcare workers and nonclinical staff at a tertiary hospital: A prevaccine census study

**DOI:** 10.1371/journal.pone.0267619

**Published:** 2022-10-27

**Authors:** Daniel Maina, Geoffrey Omuse, George Ong’ete, Patrick Mugaine, Shahin Sayed, Zahir Moloo, Reena Shah, Anthony Etyang, Rodney Adam

**Affiliations:** 1 Department of Pathology & Laboratory Medicine, Aga Khan University Hospital, Nairobi, Kenya; 2 Occupational Safety & Health, Aga Khan University Hospital, Nairobi, Kenya; 3 Aga Khan Health Services, Nairobi, Kenya; 4 Department of Internal Medicine, Aga Khan University Hospital, Nairobi, Kenya; 5 KEMRI–Wellcome Trust Research Programme, Kilifi, Kenya; Kyung Hee University School of Medicine, REPUBLIC OF KOREA

## Abstract

**Background:**

Healthcare workers and nonclinical staff in medical facilities are perceived to be a high-risk group for acquiring SAR-CoV-2 infection, and more so in countries where COVID-19 vaccination uptake is low. Serosurveillance may best determine the true extent of SARS-CoV-2 infection since most infected HCWs and other staff may be asymptomatic or present with only mild symptoms. Over time, determining the true extent of SARS-CoV-2 infection could inform hospital management and staff whether the preventive measures instituted are effective and valuable in developing targeted solutions.

**Methods:**

This was a census survey study conducted at the Aga Khan University Hospital, Nairobi, between November 2020 and February 2021 before the implementation of the COVID-19 vaccination. The SARS-CoV-2 nucleocapsid IgG test was performed using a chemiluminescent assay.

**Results:**

One thousand six hundred thirty-one (1631) staff enrolled, totalling 60% of the workforce. The overall crude seroprevalence was 18.4% and the adjusted value (for assay sensitivity of 86%) was 21.4% (95% CI; 19.2–23.7). The staff categories with higher prevalence included pharmacy (25.6%), outreach (24%), hospital- based nursing (22.2%) and catering staff (22.6%). Independent predictors of a positive IgG result after adjusting for age, sex and comorbidities included prior COVID-19 like symptoms, odds ratio (OR) 2.0 [95% confidence interval (CI) 1.3–3.0, p = 0.001], a prior positive SARS-CoV-2 PCR result OR 12.0 (CI: 7.7–18.7, p<0.001) and working in a clinical COVID-19 designated area, OR 1.9 (CI 1.1–3.3, p = 0.021). The odds of testing positive for IgG after a positive PCR test were lowest if the antibody test was performed more than 2 months later; OR 0.7 (CI: 0.48–0.95, p = 0.025).

**Conclusions:**

The prevalence of anti- SARS-CoV-2 nucleocapsid IgG among HCWs and nonclinical staff was lower than in the general population. Staff working in clinical areas were not at increased risk when compared to staff working in non-clinical areas.

## Introduction

SARS-CoV-2 infection remains a threat to public health, especially in low resource settings where vaccine coverage remains low. As of 2^nd^ March 2022, only 13.9% of the Kenyan population was fully vaccinated [1]. The positivity rate with PCR testing in the general population continues to fluctuate and was less than 1% at the start of March 2022. Admissions to hospitals reflect this fluctuation. Data on the infection rates of healthcare staff in Kenya are scanty and often do not include details of exposure risk.

The country had 7,466 infected healthcare workers (HCWs) reported as of September 2021. Etyang et al. reported seroprevalences of 43.8% (urban), 12.6% (rural) and 11.5% (rural) in three counties in Kenya [2]. The challenges facing HCWs on the continent include inadequate personal protective equipment (PPE) and limited SARS-CoV-2 testing of populations that seek medical care, which leaves workers vulnerable [3], as was especially true before the provision of SARS-CoV-2 vaccination to HCWs.

Although infections in HCWs are often attributed to occupational exposure, that is not always the case. At Aga Khan University Hospital Nairobi (AKUHN), personal protective equipment (PPE) appropriate for the level of clinical care has been routinely provided since the beginning of the Kenyan outbreak. In addition, routine tests of admitted patients were implemented. Therefore, we wished to know the level of risk to HCWs and nonclinical staff where PPE and testing are readily available.

Although liberal PCR testing of HCWs and nonclinical staff has been done at AKUHN throughout the outbreak, asymptomatic staff were not routinely tested. Since asymptomatic infections have comprised a significant percentage of infections in some series, it is possible that a significant number of staff infections have been missed [4]. The serosurvey helped address the suitability of our approaches to staff safety.

## Materials and methods

This research was a census study in which all workers at AKUHN, both hospital and contracted employees, were eligible to participate. The staff were sensitised about the study through posters, institutional email addresses and group talks. One thousand, six hundred thirty-one staff consented and were recruited in the study (>60% of the workforce). The study lasted from November 2020 to February 2021, before the implementation of COVID-19 vaccination in the hospital.

Hospital staff were categorised into five groups based on the perceived risk of COVID-19 exposure at the workplace:

❖ Clinical COVID-19 areas: COVID-19 isolation wards, Intensive Care Unit (ICU), High Dependence Unit (HDU), Accident and Emergency (A&E) triage
❖ Non-COVID-19 clinical areas: general wards, outpatient clinics
❖ Allied health: laboratory, radiology, pharmacy
❖ Support staff: catering, facilities, housekeeping
❖ Academic/Administration areas

The hospital has a relatively young workforce and there was little variance in the median age among the various staff categories.

The clinical COVID-19 and non COVID-19 clinical areas had more female participants compared to males, whereas there were more males in the allied health and support staff categories.

Using the self-declared area of residence as an estimate for income, the majority of participants belong in the middle-income category as defined by the African Development Bank (AfDB) [5].

The participants populated a self-administered questionnaire with sociodemographic details including age and area of residence, medical history including COVID-19 related symptoms and details of previous COVID-19 (PCR) testing. They also reported whether they had been in close contact with household members or workmates diagnosed with COVID-19, and whether they attended to COVID-19 patients on a frequent basis. In order to determine anti-SARS-CoV-2 IgG kinetics in those with confirmed COVID-19, the actual date of PCR testing was retrieved from the laboratory information system (LIS).

The seropositivity rate (PCR) in the country at the time of carrying out this study oscillated between 10% and 20% with the capital city reporting most of the cases with a cumulative figure of 61851 as at close of March 2021 [6].

### Specimen collection and assay

A phlebotomist collected 5–10 ml of blood in a serum separator vacutainer (Greiner Bio-One GmbH–Germany) from each participant. In all cases, blood samples were labelled with a unique study identifier, centrifuged and serum separated within 24 hours of collection.

### Laboratory testing

The antibody test was performed using a chemiluminescent assay (Abbott, USA), according to the manufacturer’s instructions and standard operating procedures. The Abbott qualitative anti-nucleocapsid CoV-2 IgG assay for SARS has a manufacturer’s stated sensitivity of 100% 14 days after the onset of symptoms and a specificity of 99.6%. Our laboratory evaluation using 54 samples from known COVID-19 patients showed a sensitivity of 86%, 14 days after the onset of symptoms, and a specificity of 100% using 20 samples before COVID-19 (unpublished data). An independent evaluation of this kit by Public Health England reported a sensitivity of 93.9% for samples ≥14 days post-symptom onset of symptoms and a specificity of 100% (Bewick et al [unpublished]).

The default result unit for the SARS-CoV-2 IgG assay is Index (S/C), with a cut-off of 1.40.

This study was conducted according to the criteria set by the declaration of Helsinki on Human research. The Aga Khan University Ethics Review Committee approved the protocol (Ref: 2020/IERC-129 v4).

Written informed consent was obtained from all participants.

### Data collection and analysis

Stata 16 (StataCorp, Texas, USA) was used to perform the statistical analysis.

The demographic and risk details of the patients were captured by filling in a guided questionnaire. Descriptive statistics are presented as medians [interquartile range (IQR)] or means (standard deviation, SD) and percentages (proportions), where appropriate. Crude seroprevalence figures were adjusted for the performance (sensitivity 86%, specificity 100%) of the nucleocapsid assay in our evaluation [7].

Using prior assumptions on which factors predict a positive antibody result and how they relate to one another, we used an online tool to draw a directed acyclic graph (DAG) to determine the minimum set of variables to adjust for the total causal effect [8, 9] (presented as S1 Fig). Associations between putative risk factors and seropositivity were analysed using logistic regression and chi-square statistics, presented here, respectively, as odds ratio (OR) with 95% confidence interval (95% CI) and chi statistics with *P*-value set at 0.05 [10, 11]. The predictor variables were: positive SARS-CoV-2 PCR result, presence of flu-like symptoms, frequent close contact with COVID-19 patients at the workplace, contact with diagnosed household members and workmates, time between the PCR and antibody test and having a positive IgG result. The odds ratios were adjusted for age, sex and comorbidities (hypertension, kidney disease and diabetes).

Antibody kinetics were evaluated for participants with documented SARS-CoV-2 PCR results and compared with the proportions of staff with positive IgG at different time points between the PCR and antibody tests.

## Results

One thousand six hundred thirty-one (1,631) staff members, both AKUHN and contracted employees, participated in the study. This figure represented 60% of the hospital workforce. AKUHN employees comprised 86.7% of the study participants and females made up 56% of the tested staff. Contracted staff were younger than AKUHN employees with the median age being 28 (interquartile range [IQR] 25–36) and 36 (IQR 31–43), respectively. More AKUHN employees reported COVID-19 like symptoms in the three months prior to the study compared with the contracted staff. The age, sex composition and risk exposure across different departments are depicted in Table 1.

**Table 1:** Characteristics of study participants.

	Total	Females n (%)	Age years: Median (IQR)	Symptomatic n (%)
**AKUHN Staff**	1414	834 (59%)	36 (31–43)	699 (49%)
**Contracted Staff**	217	81 (37%)	28 (25–36)	51 (24%)
**AKUHN Divisions**				
Nursing	347	272 (78%)	35 (30–43)	200 (58%)
Pharmacy	43	32 (74%)	35 (32–39)	22 (51%)
Outreach	117	83 (71%)	37 (33–41)	62 (53%)
Pathology	84	33 (39%)	40 (33–44)	34 (40%)
Diagnostic imaging	53	21 (40%)	34 (31–38)	22 (42%)
Medical services	466	238 (51%)	34 (30–44)	233 (50%)
Others (nonclinical)	304	155 (51%)	39 (32–45)	126 (41%)
**Contracted Staff**				
Catering	53	30 (57%)	27 (25–31)	12 (23%)
Housekeeping	103	40 (39%)	29 (25–36)	26 (25%)
Security	35	10 (29%)	30 (26–39)	4 (15%)
Others	26	1 (4%)	28 (25–34)	51 (24%)
**Risk Based (AKUHN)**				
Clinical COVID^a^	209	131 (63%)	34 (30–40)	122 (58%)
Non-COVID clinical^b^	530	374 (71%)	37 (31–44)	288 (54%)
Allied health^c^	234	108 (46%)	35 (31–42)	100 (43%)
Support staff^d^	184	84 (46%)	38 (31–44)	79 (43%)
Administration/Academic^e^	249	127 (51%)	37 (29–44)	103 (42%)

**Key:** ^**a**^isolation wards, ICU, HDU, A&E triage

^b^general wards, outpatient clinics

^c^laboratory, radiology, pharmacy

^d^Support staff: catering, facilities, housekeeping

^e^nonclinical faculty, administrative staff

The overall crude seroprevalence of SARS-CoV-2 in the study was 18.4%, and 21.4% (95% CI; 19.2–23.7) after adjustment for test performance. The seroprevalence among AKUHN staff (22.0%) was not significantly different from that of contracted workers (17.7%) (*P* = 0.193). The proportion of staff with SARS-CoV-2 antibodies varied between the different cadres within these two broad groups. Notable cadres with higher proportions of exposed personnel included pharmacy (25.6%), outreach (24%), nursing (22.2%) and catering (22.6%), as shown in Table 2.

**Table 2:** SARS-CoV-2 anti-nucleocapsid IgG antibody prevalence by participant characteristics.

	Total	Seropositive n (crude prevalence, %)	Adjusted prevalence^#^ (95% confidence interval)
**All**	1631	300 (18.4)	21.4 (19.2–23.7)
**Males**	716	126 (17.6)	20.5 (17.4–24.0)
**Females**	915	174 (19.0)	22.1 (19.3–25.2)
**Age**			
20–29	368	72 (19.6)	22.8 (18.4–27.8)
30–39	666	124 (18.6)	21.6 (18.4–26.2)
40–49	392	72 (18.4)	21.4 (17.2–25.0)
50–59	145	27 (18.6)	21.6 (15.2–30.0)
≥60	45	3 (6.7)	7.8% (2.5–21.8)
**Symptomatic (preceding 3 months)**			
Yes	750	189 (25.2)	29.3 (26.1–32.7)
No	881	111 (12.6)	14.7 (12.4–17.2)
**AKUHN Staff**	1414	267 (18.9)	22.0 (19.8–24.4)
**Contracted Staff**	217	33 (15.2)	17.7 (12.8–24.0)
**AKUHN Divisions**			
Nursing	347	77 (22.2)	25.8 (21.0–31.3)
Pharmacy	43	11 (25.6)	29.8 (14.8–40.6)
Outreach	117	28 (24.0)	27.9 (19.9–37.8)
Pathology	84	14 (16.7)	19.4 (11.7–30.5)
Diagnostic imaging	53	9 (17.0)	19.8 (10.6–34.3)
Medical services	466	78 (16.7)	19.4 (15.7–23.7)
Others (nonclinical)	304	57 (18.8)	21.9 (16.9–27.4)
**Risk Based**			
Clinical COVID^a^	209	49 (23.4)	27.2 (21.2–34.5)
Non-COVID clinical^b^	530	99 (18.7)	21.7 (18.1–25.9)
Allied health^c^	234	37 (15.8)	18.4 (13.6–24.5)
Support staff^d^	185	35 (18.9)	22.0 (16.2–29.3)
Administration/Academic^e^	259	46 (18.5)	21.5 (16.4–27.7)
**Contracted Staff**			
Catering	53	12 (22.6)	26.3 (14.3–42.1)
Housekeeping	103	14 (13.6)	15.8 (8.8–25.3)
Security	35	4 (11.4)	13.3 (3.7–31.0)
Others	26	3 (11.5)	13.4 (2.9–35.1)

**Key:** ^#^Adjusted for assay’s performance: sensitivity 86% and specificity 100%

^**a**^isolation wards, ICU, HDU, A&E triage

^b^general wards, outpatient clinics

^c^laboratory, radiology, pharmacy

^d^Support staff: catering, facilities, housekeeping

^e^nonclinical faculty, administrative staff

Seven hundred sixty-nine staff (47%) had taken a SARS-CoV-2 PCR test prior to the study, with 136 (18%) confirmed positive. Seroprevalence was 66.2% among staff with a positive PCR test and 13.5% among those with a negative result (*P*<0.001). Slightly less than half (46%) of the 1631 staff reported symptoms consistent with COVID-19 in the previous 3 months prior to the study. The most prevalent symptoms were headache (52%), sneezing (51%), cough (42%) and fever (16%). Adjusted seroprevalence among symptomatic and asymptomatic staff was 29.3% and 14.7%, respectively (*P*<0.001).

### Predictors of a positive anti-SARS-CoV-2 nucleocapsid IgG test

Having previously tested for COVID-19 (PCR) irrespective of test result increased the odds of testing positive with the antibody test, OR 1.7 (95% CI: 1.3–2.2, *P*<0.001) (Table 3). The odds were even higher in those who had tested positive with PCR (OR 12.5 95% CI: 8.2–19.1, *P*<0.001). This also applied to those who had experienced flu-like symptoms in the previous 3 months prior to enrollment in the study, OR 2.3 (95% CI 1.8–3.0, *P*<0.001); antibody test performed 2–4 weeks after the PCR test, OR 1.6 (95% CI 1.02–2.54, *P* = 0.041); or reported daily contact with COVID-19 patients, OR 1.5 (95% CI 1.2–2.0, *P* = 0.002); or worked in a clinical COVID-19 designated area, OR 1.4 (95% CI 1.0–2.0), *P* = 0.044).

**Table 3:** Risk factors for a positive anti-nucleocapsid IgG result.

	SARS-CoV-2 IgG Positive n (%)	Odds Ratio (95% CI)	*P*-value
**Previously tested for SARS-CoV-2 (PCR)**			
**Yes**	175 (22.8)	1.7 (1.3–2.2)	<0.001
**No**	124 (14.5)		
**Time interval between PCR and serology test**			
<2 weeks	25 (23.6)	1.1 (0.6–1.7)	0.855
2–4 weeks	32 (30.8)	1.6 (1.02–2.54)	0.041
1–2 months	37 (25.7)	1.2 (0.8–1.8)	0.375
>2 months	80 (19.7)	0.7 (0.48–0.95)	0.025
**COVID-19 PCR result**			
Positive	90 (66.2)	12.5 (8.2–19.1)	<0.001
Negative	85 (13.5)		
**Flu-like symptoms past 3 months**			
Yes	189 (25.2)	2.3 (1.8–3.0)	<0.001
No	111 (12.6)		
**Univariate analysis of individual symptoms**			
Cough	93 (29.6)	2.3 (1.7–3.0)	<0.001
Sneezing	92 (24.2)	1.6 (1.2–2.1)	0.001
Fever	50 (42.4)	3.7 (2.5–5.5)	<0.001
Running nose	87 (24)	1.6 (1.2–2.1)	0.002
Chest pain	29 (26.6)	1.7 (1.1–2.6)	0.023
Headache	102 (26)	1.8 (1.4–2.4)	<0.001
Loss of smell	59 (67.8)	11.4 (7.1–18.2)	<0.001
Multivariate analysis symptoms^$^			
Cough		1.7 (1.2–2.4)	0.004
Fever		1.7 (1.1–2.7)	0.029
Loss of smell		8.6 (5.2–14.2)	<0.001
**Household contacts or workmates diagnosed with covid**			
Yes	163 (19.6)	1.2 (0.9–1.5)	0.22
No	137 (17.2)		
**Work involves frequent (daily) close contact with COVID-19 patients**			
Yes	190 (21.1)	1.5 (1.2–2.0)	0.002
No	110 (15.1)		
**Hospital Location Risk (clinical COVID)**			
Yes	49 (23)	1.4 (1.0–2.0)	0.044
No	251 (18)		

^$^Covariates included: cough, headache, fever, sneezing, running nose, headache and loss of smell

Univariate analysis showed a significant association between the most prevalent symptoms cough, headache, fever, sneezing, coryza and loss of smell with the positive Covid-IgG test. However, multivariate analysis of the symptoms revealed an independent association with only cough, fever and loss of smell.

In the multivariate model for predictors, a positive PCR result, working in a clinical COVID-19 area and flu-like symptoms predicted seropositivity. However, reporting interacting daily with COVID-19 patients, or contact with diagnosed household members and workmates, and the time interval between PCR and antibody tests did not achieve statistical significance (Table 4).

**Table 4:** Predictors of a positive anti-nucleocapsid IgG result.

	Univariate		Multivariate Model^a^	
	Odds Ratio (95% CI)	P-value	Odds Ratio (95% CI)	*P*-value
SARS-CoV-2 PCR+ results	12.5 (8.2–19.1)	<0.001	12.0 (7.7–18.7)	<0.001
Flu-like symptoms past 3 months	2.3 (1.8–3.0)	<0.001	2.0 (1.3–3.0)	0.001
Work involves frequent (daily) close contact with COVID-19 patients	1.5 (1.2–12.0)	0.004	1.1 (0.7–1.7)	0.782
Contact with diagnosed household members or workmates	1.2 (0.9–1.5)	0.222	0.7 (0.5–1.1)	0.161
Hospital Location Risk (clinical COVID)	1.4 (1.0–2.0)	0.044	1.9 (1.1–3.3)	0.021
Time interval (2–4 weeks) between PCR and antibody test	1.6 (1.0–2.5)	0.041	1.3 (0.7–2.2)	0.413

^a^Model included: SARS-CoV-2 PCR+ result, flu-like symptoms, frequent contact with COVID-19 patients, contact with diagnosed household members or workmates, working in a COVID-19 designated area, and time interval between PCR and antibody tests. Adjusted for age, sex and comorbidities

### COVID-19 IgG dynamics

**Fig 1:**
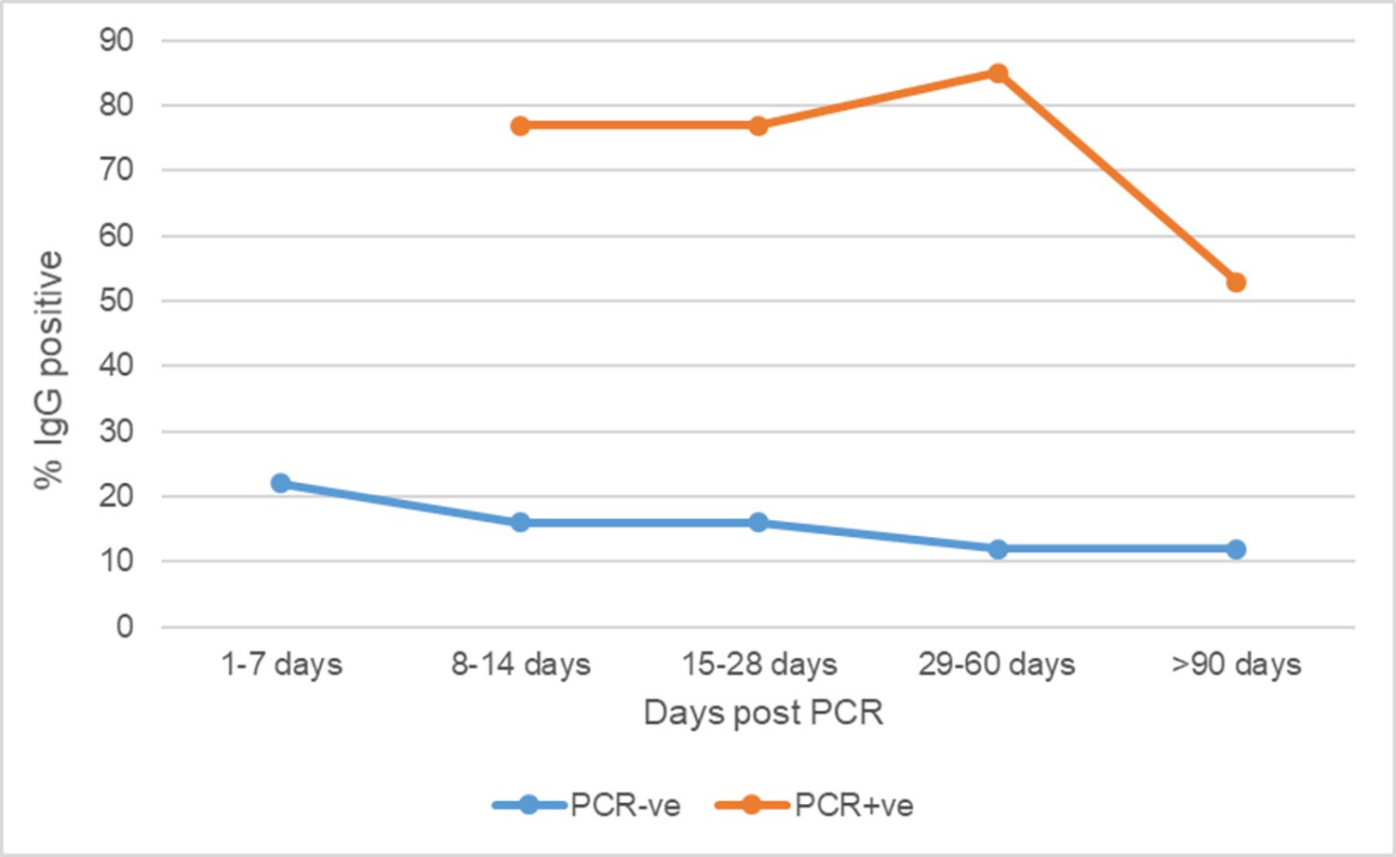
Proportion of HCWs and nonclinical staff with positive IgG antibody over time after PCR test.

**Fig 2:**
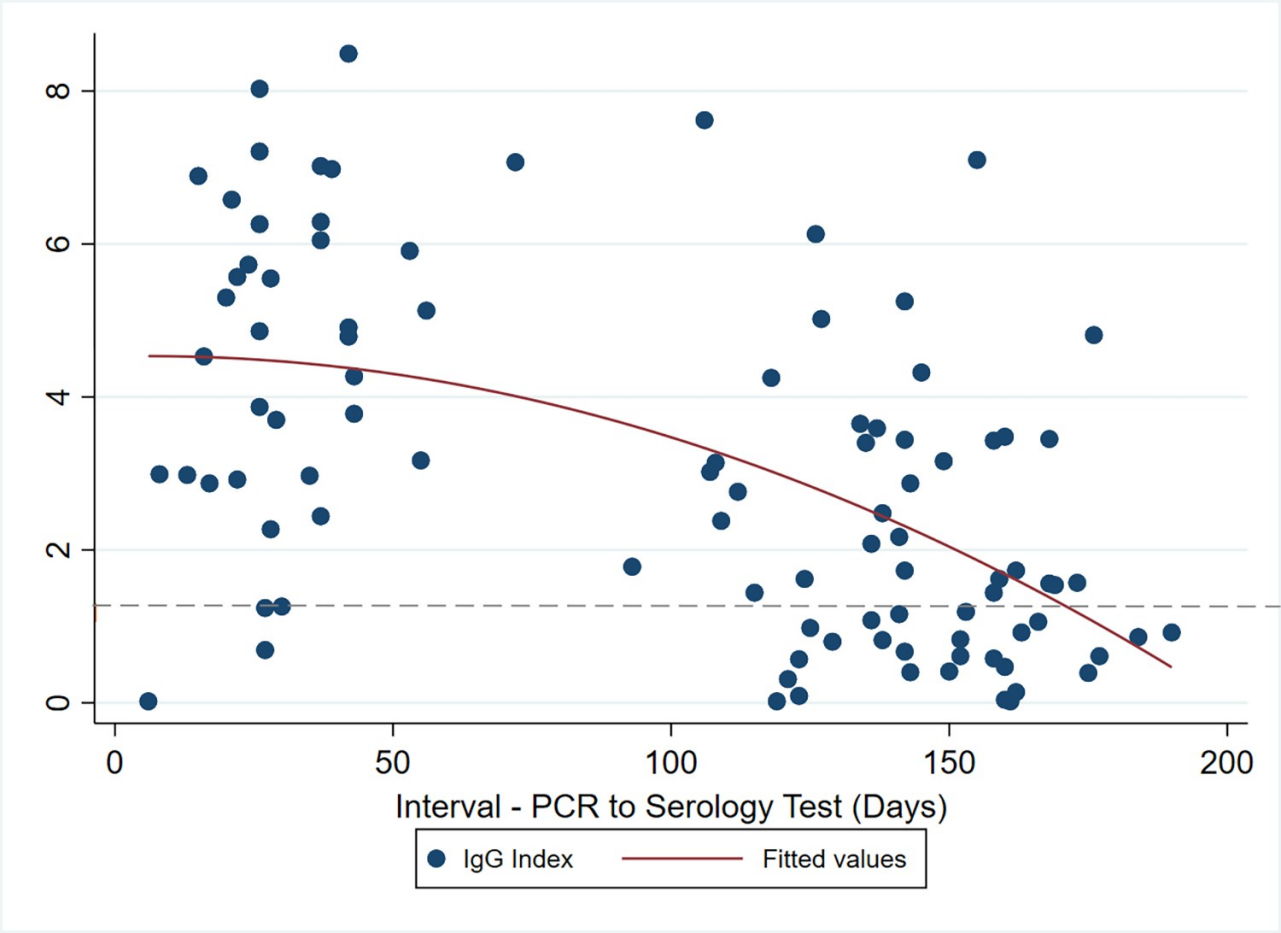
Antibody index at various time points post-SARS-CoV-2-19 PCR+ test.

The seroprevalence in participants who tested positive for SARS-CoV-2 by PCR peaked 1–2 months following the PCR test and sharply declined thereafter (Figs 1 and 2). The odds of testing antibody positive were lowest if performed more than 2 months after PCR, OR 0.7 (CI 0.48–0.95). The PCR negative group had a steady but slow decline in seroprevalence throughout the same period.

The dashed horizontal line is the assay cut-off index (1.4)

## Discussion

We determined the seroprevalence and kinetics of SARS-CoV-2 nucleocapsid IgG antibodies in HCWs and nonclinical staff prior to the advent of the COVID-19 vaccine when little information was available on the clinical implications (immunity) of seroconverting. This was also before any variant of concern had been reported in the country. The adjusted seroprevalence of 21% was well below any reported in a public urban hospital (43.8%) in Nairobi (2). Whether the strict COVID-19 prevention measures enforced early in AKUHN contributed substantially to the relatively lower seroprevalence has not been determined. Hospitals in rural areas had lower rates reflecting low community transmission (2). Early in the COVID-19 pandemic, Nairobi was the epicentre of the infection, which later spread to other parts of the country.

Elsewhere on the continent, seroprevalence among HCWs ranged from 0% to 45.1%, with the highest prevalence in Nigeria [12].

Contrary to previous assumptions, there was no significant difference in seroprevalence between frontline staff (high-risk work-based exposure) and staff working in nonclinical areas (low-risk work exposure), suggesting that most of the SARS-CoV-2 infections occurred in the community. During the same period, a study conducted in India reported similar figures between HCWs and the general public (25.6% versus 23.2%) [13]. Here in Kenya, a study reported a higher prevalence (61.8%) among blood donors than the prevailing seroprevalence among HCWs in Nairobi for the period January through March 2021 [14], reflecting the high community transmission prevalent during the study period.

The seroprevalence across the age groups was similar for staff under 60 years of age and then decreased for age ≥60. Some studies have found increased seropositivity with increasing age for some age brackets [15, 16]. Pharmacy and catering staff had the highest prevalence, possibly explained by confined working spaces with close contact between the staff in the two areas.

Testing positive for SARS-CoV-2 by PCR, working in a COVID-19 designated area and having flu-like symptoms independently predicted a positive SARS-CoV-2 antibody result. The three symptoms that independently predicted a positive antibody result were loss of smell, followed by fever and cough (adjusted OR 8.7, 1.7, 1.7, respectively). Other studies have reported similar symptoms among HCWs elsewhere [17, 18].

Symptomatic staff had mild COVID-19 disease, none of which required hospitalisation. The hospital workforce is relatively young (mean age of 37 years) and has few comorbidities, which could partly explain the mild COVID-19 disease seen in the facility.

Correct interpretation of serology test results requires a proper understanding of the antibody dynamics, as demonstrated in our findings. Testing for nucleocapsid IgG antibodies three months after SARS-CoV-2 infection is likely to miss a significant proportion of exposed people. Only 20% of HCWs with confirmed infection in our study were positive for IgG antibodies at three months and beyond. Van Elslande et al. reported that anti-nucleocapsid IgG antibody levels of SARS-CoV-2 steadily decreased after 2 months up to 8 months after PCR, while another study showed a decline in positivity starting at 6 months onward after the onset of COVID-19 symptoms or testing [19, 20].

All participants in our study had mild disease or were asymptomatic and the kinetics described here may not apply to those with severe COVID-19 disease, which has been associated with earlier and higher antibody levels compared with asymptomatic infections [21, 22].

Our study has several limitations. Apart from diagnosed household contacts, we did not determine other potential sources of outside work exposure such as indoor gatherings and mode of transport to work (public versus private), This has the potential to mask the effect of exposure risk between the high and low risk hospital locations.

The study was a survey in which approximately 60% of the targeted workforce volunteered. We were not able to determine whether those who did not participate differed from the participants thus self-selection bias could have arisen.

Recall bias and underreporting may have affected reporting of previous symptoms, contact with diagnosed household members and workmates, and comorbidities.

Lastly, this was a cross-sectional study in which the antibody test was performed only once and could have missed participants with recent infections or decayed antibody response. This could have resulted in underestimating the true seroprevalence.

## Conclusions

The prevalence of anti- SARS-CoV-2 nucleocapsid IgG among HCWs and nonclinical staff was high but lower than in the general population. There were no statistically significant differences in seroprevalence between the clinical areas and the rest of the hospital. Independent predictors of a seropositive result were a positive SARS-CoV-2 PCR result, working in a COVID-19 designated area and flu-like symptoms in the 3 months prior to joining the study. Seropositivity peaked 2 months after a positive PCR test and then declined.

## Supporting information

S1 Fig Directed acyclic graph to model the association between SARS-CoV-2 exposure and anti-SARS-CoV-2 IgG detection. Biasing paths are indicated by red arrows, causal path are indicated by green arrows. Ancestors of outcome are drawn in blue, ancestors of exposure and outcome are drawn in red. Ancestors of SARS-CoV-2 infection depicted in green and unobserved effects in grey. Minimal sufficient adjustment sets for estimating the total effect SARS-CoV-2 exposure on IgG detection: Age, Sex and Comorbidities.
(TIF)

S1 Data (XLS)
