# Supplementary figures and images for: Seroprevalence, correlates and kinetics of SARS-CoV-2 nucleocapsid IgG antibody in healthcare workers and nonclinical staff at a tertiary hospital: A prevaccine census study
Source: PLoS One. 2022 Oct 27;17(10):e0267619. doi: 10.1371/journal.pone.0267619 (PMC9612503; doi:10.1371/journal.pone.0267619)

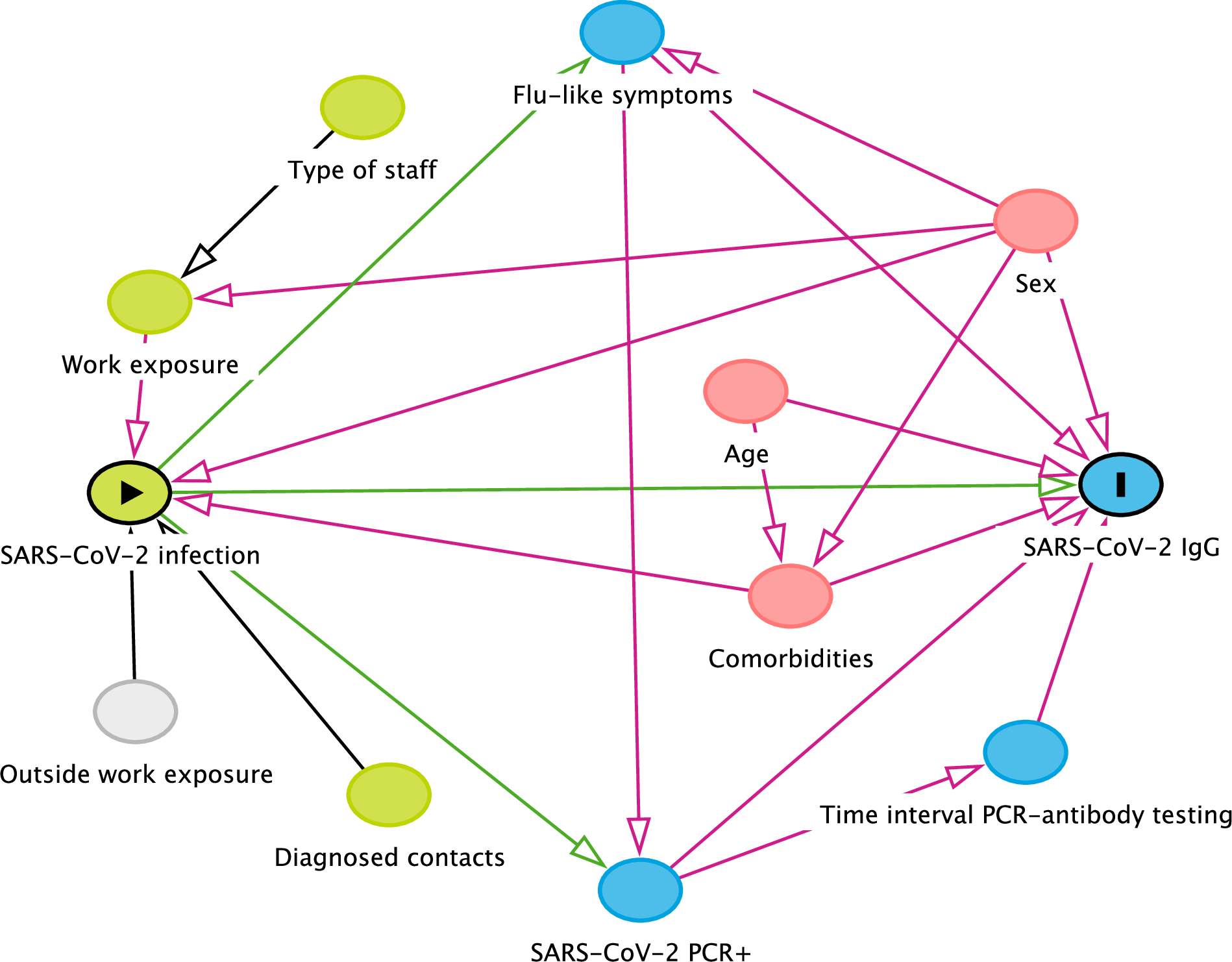

Supplement: S1 Fig — Biasing paths are indicated by red arrows, causal path are indicated by green arrows. Ancestors of outcome are drawn in blue, ancestors of exposure and outcome are drawn in red. Ancestors of SARS-CoV-2 infection depicted in green and unobserved effects in grey. Minimal sufficient adjustment sets for estimating the total effect SARS-CoV-2 exposure on IgG detection: Age, Sex and Comorbidities. (TIF) [file pone.0267619.s001.tif]
